# Content, context, cues, and demeanor in deception detection

**DOI:** 10.3389/fpsyg.2022.988040

**Published:** 2022-08-08

**Authors:** Timothy R. Levine

**Affiliations:** Department of Communication Studies, University of Alabama at Birmingham, Birmingham, AL, United States

**Keywords:** content, context, cues, demeanor, deception

## Introduction

Knowing the truth matters. Therefore, improving truth and deception detection is a worthy scholarly endeavor with important applications throughout society.

A large but fragmented literature exists on the topic of deception detection spanning a variety of academic disciplines (Vrij, 2008; Levine, 2020; Denault et al., 2022). Much of the research has an applied focus, aiming to improve lie detection in a particular setting or genre such as criminal investigation, fraud prevention, or political disinformation.

The need for improved lie detection is urgent and real. Research has shown that people generally over-estimate their lie detection ability (DePaulo et al., 1997) and that people are poor lie detectors (Bond and DePaulo, 2006).

Contemporary approaches to lie detection often involve trying to isolate cues associated with honesty and deception. I call these approaches “cue theories” (Levine, 2020). The core idea underlying the cue theories is that the observation of the right cues under the right conditions can probabilistically improve lie detection. I doubt the diagnostic value of cues in assessing the veracity of specific instances of communication. This, however, is an argument I make elsewhere (Levine, 2018, 2020). Here, I focus on some conception distinctions relevant to the current topic from a communication perspective.

## Definitions and distinctions

Different authors sometimes use the same words to mean different things. For example, some of the “cues” listed in the DePaulo et al. (2003) meta-analysis would not be cues according to my definition. As I think of them, cues are specific observable behaviors. They can be nonverbal, such as gaze aversion, finger movements, or speech errors, or they can be verbal such as the number of details or type of pronouns. Cues can be counted, timed, or otherwise objectively measured. They can be expressed in quantities.

Cues, however, do not travel alone. During segments of communication, cues are highly intercorrelated with other cues (Levine et al., 2011). This is also how they are perceived. Statistically, treating cues as if they are independent from one another (e.g., as in a lens model; Hartwig and Bond, 2011) potentially violates statistical assumptions and risks spurious interpretations of results. Pragmatically, training communicators to focus on specific behaviors might lead to tunnel vision and be disruptive to conversational flow (see Vrij et al., 2022 for additional concerns).

I call constellations of inter-correlated cues “demeanor” (Levine et al., 2011). Perceptions of confidence, friendliness, extroversion, and authenticity are examples of demeanors one can give off. Various demeanors can be quantitatively scaled either as clusters of coded cues used to create an index, or as global impressions by observers. Interestingly, in DePaulo et al.'s (2003) meta-analysis, four out the top five most diagnostic indicators were demeanors (immediacy, discrepant, uncertain, nervous) and only one was a cue (details). Nevertheless, I argue that demeanors lead to systematic and predictable errors in human veracity judgments because there are a substantial number of individual communicators whose demeanors are “mismatched” with their internal states (Levine et al., 2011). For example, an honest person on the autism spectrum might come off as deceptive because of their demeanor (Lim et al., 2021).

Both cues and demeanor can be further distinguished from communication content. Content involves the meaning of what is said. Content is not a cue. It can't be counted. Meanings are fundamentally qualitative in nature. Further, meanings can be highly contextual. Hall (1976) famously advanced the idea of high and low context communication. In low context communication, one needs only to know the language to understand. High context communication, however, requires background knowledge to understand. An example is satire. To understand satire, you must know what is being satirized.

Details provide an example of my distinction between cues and content. Just counting the number of details in a verbal account is a cue. Verifiable details (Nahari et al., 2014; Verschuere et al., 2021) have an element of content to them, but they are still counted and are thus also a cue. It does not matter what the detail is; only if it is a detail, and if it is, in principle, subject to being checked or not. In contrast, when viewing details as communication content, we consider what each of the details are. Do they make sense given the context? How do the fit with other known details? Any detail or set of details may or may not be diagnostic of honesty-deceit depending on what the detail means in the context in which it is provided and how it fits with other knowledge.

Plausibility is a second example. Unlike DePaulo et al. (2003) and Vrij et al. (2020), I do not think of plausibility as a cue, but rather as a scalable attribute of content (understanding what is said in context and then assessing its typicality or probability of occurrence). Further, as an aspect of communication content, meaningfully assessing plausibility is different for low and high context communication and depends on the relevant contextual knowledge of the person assessing plausibility.

A deep understanding of context is required for assessing content in high-context communication. For example, consider the results of a deception detection article reporting 36% accuracy. Obviously, that is poor accuracy (a low context reading requiring little prior knowledge). But how plausible is it? I know, for example, that it is three standard deviations below the meta-analytic mean (Bond and DePaulo, 2006), and that there are few theoretical mechanisms that produce below-chance accuracy. Knowing the literature contextualizes the claim. As this example illustrates, assessments of plausibility can vary depending on the knowledge and expertise of those doing the assessment.

Thus, when I think of context, I am not just thinking in terms of categories of situations or applications (e.g., investigative police interviewing vs. political journalism). Nor do I think about context as fact-checking and plausibility (cf. Vrij et al., 2020) which I see as potentially diagnostic aspects of communication content. Context involves what someone needs to know to understand and make sense out of what is said, and to think critically about content. Amongst other things, it involves knowing what was said in prior and subsequent utterances, the specifics of the situation in which the communication occurs, the personal backgrounds of the communicators, their idiosyncrasies, and the (sub-)culture(s) of the communicators. Thus, having knowledge of communication context is an enabling factor (moderator) for fact-checking or assessing the plausibility of communication content. Fact-checking and plausibility assessment require context, but they are not types of contexts.

## Application

My opinion regarding interviewing to assess veracity (besides building rapport and asking non-leading open-ended questions; both good practices in my opinion) is that it is wise to ignore cues and demeanor (cf. Masip and Herrero, 2015). Confident, friendly, extroverts who provide detailed accounts are not always honest. Similarly, people with poor memories or nonobvious cognitive impairments might be honest. As a personal example, I lack visual memory. I cannot honestly give you a visually detailed description of a true event I witnessed. I am also dyslexic. When I transpose things, the correct interpretation may be as a sign of dyslexia, but you would know that unless you asked or you knew me well enough to know.

Instead, I advocate careful listening to communication content. Understand that content in context—the deeper and richer the contextualization, the better. Then, apply critical thinking. The more that is known about the context, the more potentially valuable communication content becomes (Blair et al., 2010; Reinhard et al., 2011). Interviewers should avoid going into interviews cold. My advice is to investigate first and then interview. Not only does this make the strategic use of evidence possible (Hartwig et al., 2006), but it also provides more context. Having context enables asking the right questions as well as a better understanding of the answers. Good questions prompt answers that can be fact checked or evaluated for plausibility. If verifiable details are provided, check them (Blair et al., 2018). For details that are not yet checkable, perhaps additional evidence with be uncovered over time. Assess plausibility based on contextual knowledge, and revisit and update evaluations of plausibility as new information is acquired. Unlike cues, plausibility is not a static, stable, or fixed quality of a message. It changes depending on what else is known. Think of veracity assessment as an ongoing process, not a fixed or one-time judgment.

## Conclusion

Useful distinctions can be made between cues, demeanors, and communication content. Understanding communication content requires knowledge of context, especially in high-context communication. A richer and deeper understanding of communication-specific context unlocks the utility of communication content in distinguishing honest and deceptive statements.

## Author contributions

TL conceptualized and wrote this essay.

## Conflict of interest

The author declares that the research was conducted in the absence of any commercial or financial relationships that could be construed as a potential conflict of interest.

## Publisher's note

All claims expressed in this article are solely those of the authors and do not necessarily represent those of their affiliated organizations, or those of the publisher, the editors and the reviewers. Any product that may be evaluated in this article, or claim that may be made by its manufacturer, is not guaranteed or endorsed by the publisher.
